# Risk of Bleeding and Stroke with Oral Anticoagulation and Antiplatelet Therapy in Patients with Atrial Fibrillation in Taiwan: A Nationwide Cohort Study

**DOI:** 10.1371/journal.pone.0125257

**Published:** 2015-04-29

**Authors:** Pei-Chun Chen, Gregory Y. H. Lip, Grace Yeh, Hung-Ju Lin, Kuo-Liong Chien

**Affiliations:** 1 Clinical Informatics and Medical Statistics Research Center, Chang Gung University, Tao-Yuan, Taiwan; 2 University of Birmingham Centre for Cardiovascular Sciences, City Hospital, Birmingham, United Kingdom; 3 National Taiwan University Health Data Research Center, Taipei, Taiwan; 4 Institute of Epidemiology and Preventive Medicine, College of Public Health, National Taiwan University, Taipei, Taiwan; 5 Department of Internal Medicine, National Taiwan University Hospital, Taipei, Taiwan; University of Florida, UNITED STATES

## Abstract

**Background:**

Data on the use of oral anticoagulation (OAC) and antiplatelet therapy and the risk of bleeding and stroke amongst Asian patients with atrial fibrillation (AF) are limited. We investigated the risks of bleeding and stroke with use of oral anticoagulation (OAC) and antiplatelet therapy as mono- or combination therapy, in patients with AF from a Chinese nationwide cohort study.

**Methods:**

We studied a cohort of 10384 patients (57.2% men, age 67.8 ± 13.2 yrs) between 1999 and 2010 from the National Health Insurance Research Database in Taiwan. Records of prescriptions were obtained during follow-up. The main outcome was a recurrent stroke during the follow-up period. Time-dependent Cox proportional hazards models were used for this analysis.

**Results:**

We documented 1009 events for bleeding, as well as 224 hemorrhagic stroke and 1642 ischemic stroke events during a median 3.2 (interquartile range, 1.05-6.54) years’ follow-up. Compared with warfarin users, patients with antiplatelet therapy had a lower risk of bleeding (adjusted relative risk [RR], 0.59, 95% confidence interval [CI], 0.49-0.71, p<0.001) whilst combination therapy had a non-statistically significant higher bleeding risk (RR, 1.33, 95%, 0.91-1.94, p = 0.20). Patients on antiplatelet monotherapy had a similar risk for ischemic stroke compared with OAC (RR 1.05, 95% CI, 0.89-1.25, p = 0.50), whilst those on combination therapy had a significantly higher risk (RR 1.90, 95% CI, 1.34-2.70, p<0.001).

**Conclusion:**

In a national representative cohort, antiplatelet therapy had no significant difference in ischemic stroke risk to warfarin. For bleeding, aspirin had a lower risk compared to warfarin. This may reflect poor anticoagulation control, highlighting important missed opportunities for improved stroke prevention, especially in countries where anticoagulation management is suboptimal.

## Introduction

Atrial fibrillation (AF) is the most frequent cardiac arrhythmia, with the prevalence increasing progressively with age [1]. A national survey based on health insurance data in Taiwan showed hospitalizations due to AF increased from 91 per 100,000 to 150 per 100,000 between 1997 and 2002 [2,3]. AF is an important risk factor for stroke, which confers a major healthcare burden on acute or long-term medical care, as well as on patient disability[4,5].

Various stroke risk stratification schemes, including the CHADS_2_[6] and CHA_2_DS_2_-VASc[7] scores for stroke risk, and the HAS-BLED score for bleeding risk, have been used to aid risk prediction in AF although some ethnic and gender differences may be evident [8–10].

Stroke prevention in AF requires the use of oral anticoagulation (OAC) with antiplatelet therapy only having a weak efficacy[11]. Both OAC and antiplatelet drugs confer an increased risk of bleeding, which is accentuated by its combined use amongst AF patients[12–14]. However, the balance between efficacy and safety with OAC also depends on the quality of INR control, as reflected by the average time in therapeutic range (TTR) [15,16], with the recommended TTR being >70%[17]. In many countries, the average TTR is poor, and in a recent randomized trial, the average TTR in Taiwan was only 44%[18].

One small hospital based study from Beijing, China reported that stroke and bleeding rates were not different between warfarin, aspirin and untreated patients, reflecting that many warfarin-treated patients did not have regular access to anticoagulation monitoring and offering opportunities for improved stroke prevention with the novel OAC drugs[19]. We are unaware of any large cohorts comparing stroke and bleeding risk in the Far East, where OAC control with warfarin may be suboptimal. If the findings from the paper by Guo et al[19] were replicated in a large nationwide cohort, this would have major implications for missed opportunities for stroke prevention in AF, as well as important healthcare cost and public health implications.

In this study, we investigated stroke and bleeding rates in a national representative population amongst AF patients treated with OAC (warfarin), antiplatelet drugs as monotherapy and as combination therapy, compared to no antithrombotic therapy. Second, we assessed event rates according to antithrombotic therapy use, by stroke and bleeding risk strata, using established risk scoring systems, that is, CHADS_2_ and CHA_2_DS_2_-VASc for stroke risk, and the HAS-BLED score for bleeding risk.

## Methods

### Data sources and searches

This study used a subset of National Health Insurance Research Database (NHIRD), the Longitudinal Health Insurance Database of 2000, which contains claims data of a randomly sampled cohort of one million people enrolled in the Taiwan National Health Insurance program during 1996–2000. The health insurance program has covered 99% or more of Taiwanese population and contracted more than 90% of healthcare institutions in Taiwan. We obtained the original claim data that included inpatient records, ambulatory care records, contracted pharmacies records and registries for beneficiaries. The Institutional Review Board in National Taiwan University Hospital approved the study protocol. The patient records/information was anonymized and de-identified prior to analysis and the informed consent was waived.

Patients had a first diagnosis for AF (the index admission) during 1999–2010 was considered as follows: the first diagnosis for AF: the presence of at least one inpatient claim with an ICD-9-CM code 427.3 in any one of up to 5 diagnostic codes or at least two outpatient claims with an ICD-9-CM code 427.3 in any one of up to 3 diagnostic codes. The date of diagnosis was defined as the index date. The accuracy of the ICD-9-CM codes for AF has been validated by medical chart review [20]; of patients who had an AF diagnosis by ICD-9-CM codes and used medications that might be prescribed to AF patients, 98% and 96% with AF recorded by either electrocardiogram or 24-hour Holter monitoring in a medical center and community teaching hospital, respectively. The exclusion criteria included the following: age at index date <18 years, patients who died during the index admission, patients with rheumatic heart disease (ICD-9-CM codes 393–398 in any one of the five positions on at least one inpatient claims or in any one of the three positions on at least two outpatient claims), and patients who died during the index admission.

We defined antithrombotic drug exposure into 8 groups based on prescription after the index date: (i) OAC (essentially warfarin) monotherapy; (ii) aspirin monotherapy; (iii) clopidogrel monotherapy; (iv) aspirin + clopidogrel; (v) warfarin + aspirin; (vi) warfarin + clopidogrel; (vii) warfarin + aspirin +clopidogrel; and (viii) no antithrombotic therapy (ie. never users of warfarin, aspirin, clopidogrel). We calculated the usage according to the prescription history: ‘monotherapy’ was defined as group (1) to group (iii); ‘combination therapy’ was defined as group (iv) to group (vii). We defined ‘continuous use’ if the number of discontinued days was less than 7 days.

We defined the comorbidity histories, drug usage codes according to ICD-9-CM codes as the S1 Table. The definitions for the CHADS_2_, CHA_2_DS_2_-VASc and HAS-BLED scores were listed according to the clinical status within 2 years of the index date (S2 Table).

### Outcome Measurements

The follow-up duration began on the index date (or discharge date of index admission) and lasted until the “outcome” diagnosis, withdrawal from National Health Insurance, or December 31, 2010, whichever came first. We defined the following outcomes: (i) bleeding was defined as hospital admission for bleeding, including gastrointestinal, intracranial, urinary tract and airway bleeding episodes in the follow-up duration; (ii) hemorrhagic stroke: admission for hemorrhagic stroke (ICD9CM codes 431–432) in the follow-up duration; and (iii) ischemic stroke defined as admission for ischemic stroke (ICD9CM codes 433–437) in the follow-up duration.

### Statistical analysis

Demographic and clinical characteristics of the study participants were listed according to various therapy groups and were compared by ANOVA for continuous variables and by the chi-square test for categorical data. We calculated the incidence rate (per 1000 person-years) by dividing numbers of events of bleeding and stroke events with person-years of exposure to each group. Due to time varying nature of drug exposure, we defined the duration of specific drug exposure as days of use for each prescription from database at ambulatory care, and contracted pharmacies, but not at inpatient records because data of days of use of each prescription were not available.

We used multivariable models to estimate the relative risk (RR) and 95% confidence interval (CI) by Cox proportional hazards model with drug exposure as a time-varying covariate to assess the association between various groups and bleeding as well as stroke events. We used the single anticoagulant group (warfarin monotherapy) as the reference group[13] and adjusted for age, gender and comorbidity status, including history of ischemic heart, hypertension, ischemic stroke, heart failure, diabetes, liver disease, renal failure, malignancy, bleeding, and drug history of aspirin, warfarin and clopidogrel usage which were defined before index atrial fibrillation date. Moreover, we calculated the numbers and crude incidence rates of ischemic stroke and bleeding events according to the categories of CHADS_2_, CHA_2_DS_2_-VASc and HAS-BLED scores among various drug exposure groups to evaluate potential effects of drug treatment on the impact of risk stratification. All data analyses were performed using SAS version 9.3 (SAS Institute Inc., Carey, NC).

## Results

We included 10384 patients (57.2% male; mean age 67.8 years) with first-time AF diagnosis and live at discharge between 1999 and 2010 [Table 1]. Compared with those treated with warfarin therapy, patients taking aspirin and clopidogrel were older and more likely to be male, and to have a history of ischemic heart disease, heart failure, hypertension, ischemic stroke, diabetes, renal failure, and history of bleeding, including gastrointestinal, intracranial and urinary track bleeding, and amiodarone use.

**Table 1:** Study cohort and comorbidities at 2 years before index AF.

Name	Total	Warfarin	Aspirin	Clopidogrel	Aspirin+ Clopidogrel	Warfarin + Aspirin	Warfarin + Clopidogrel	Warfarin + Aspirin+ Clopidogrel	None
Patients, total n (%)	10384	1941(18.7)	7237(12.5)	1298 (12.5)	1369 (13.2)	1277 (12.3)	205 (2.0)	196 (1.9)	2408(23.2)
Age, yr	67.8(13.2)	67.4(12.1)	68.8(10.2)	72(12.0)	70.8(10.5)	68.3(10.7)	69.5(10.0)	68.2(9.1)	65.1(12.2)
Men, n (%)	5915(57.2)	1107 (57.0)	4168(57.6)	756(58.2)	845(61.7)	755(59.1)	124(60.5)	132(67.3)	1333(55.4)
Women, n (%)	4424(42.8)	834 (43.0)	3069(42.4)	542(41.8)	524(38.2)	522(40.9)	81(39.5)	64(32.7)	1075(44.6)
Comorbidities, n (%) ^a^									
Acute MI	37(0.4)	9(0.5)	25(0.4)	9(0.7)	16(1.2)	5(0.4)	2(1.0)	2(1.0)	6(0.3)
Ischemic heart ^b^	789 (7.6)	120(6.2)	574(7.9)	184(14.2)	207(15.1)	99(7.8)	28(13.7)	27(13.8)	148(6.2)
Heart failure ^c^	320(3.1)	78(4.0)	291(4.0)	74(5.7)	77(5.6)	48(3.8)	8(3.9)	12(6.1)	133(5.5)
Hypertension	1667(16.5)	238(12.3)	1136(15.7)	286(22.0)	307(22.4)	177(13.9)	34(17.0)	41(20.9)	401(16.7)
Ischemic stroke	634(6.1)	97(5.0)	427(5.9)	109(8.4)	102(7.5)	82(6.4)	19(9.3)	18(9.2)	158(6.7)
Diabetes	966(9.3)	125(6.4)	622(8.6)	197(15.2)	197(14.4)	88(6.9)	23(11.2)	31(15.8)	254(10.6)
Liver disease	267(2.6)	32(1.7)	164(2.3)	28(2.2)	23(1.7)	16(1.3)	3(1.5)	1(0.5)	94(3.9)
Renal failure	210(2.0)	21(1.1)	119(1.6)	37(2.9)	39(2.9)	8(0.6)	2(1.0)	3(1.5)	76(3.2)
Malignancy	311(3.0)	21(1.1)	136(1.9)	34(2.6)	24(1.8)	9(0.7)	3(1.5)	3(1.5)	150(6.2)
Bleeding	203(2.0)	23(1.2)	112(1.6)	28(2.2)	31(2.3)	13(1.0)	4(2.0)	2(1.0)	83(3.5)
Gastrointestinal bleeding ^d^	40(0.4)	6(0.3)	21(0.3)	7(0.5)	8(0.6)	2(0.0)	1(0.5)	1(0.5)	18(0.8)
Gastrointestinal bleeding ^e^	92(0.9)	11(0.6)	49(0.7)	16(1.2)	17(1.2)	6(0.5)	3(1.5)	1(0.5)	40(1.7)
Intracranial bleeding	54(0.5)	6(0.3)	30(0.4)	8(0.6)	9(0.6)	5(0.4)	1(0.5)	0(0)	23(1.0)
Urinary tract bleeding	44(0.4)	4(0.2)	24(0.3)	4(0.3)	5(0.4)	1(0.1)	0(0)	0(0)	17(0.7)
Airway bleeding	13(0.1)	2(0.1)	9(0.1)	0(0)	0(0)	1(0.1)	0(0)	1(0.5)	3(0.1)
Previous antithrombotic treatment, n (%)^f^									
Aspirin	5650(54.4)	1071(55.2)	4698(64.9)	800(61.6)	957(69.9)	879(68.8)	111(54.2)	129(65.8)	599(24.9)
Warfarin	946(9.1)	726(37.4)	574(7.9)	127(9.8)	112(8.2)	379(30.0)	59(28.8)	62(31.6)	54(2.2)
Clopidogrel	773(7.4)	112(5.8)	465(6.4)	361(27.8)	328(24.0)	79(6.2)	46(22.4)	36(18.4)	100(4.2)
Antiarrhythmic drug treatment, n (%)									
Amiodarone	2502(24.1)	360(18.5)	1509(20.9)	295(22.7)	241(17.6)	164(12.8)	25(12.2)	14(7.1)	751(31.2)
Propafenone	643(6.1)	310(16.0)	233(3.2)	179(13.8)	356(26.0)	263(20.6)	104(50.7)	13(6.6)	79(3.3)
Sotalol	553(5.3)	142(7.3)	154(2.1)	123(9.5)	234(17.1)	347(27.2)	98(47.8)	9(4.6)	99(4.1)

a: Comorbidities(Acute MI, Ischemic heart ^b^ Heart failure ^c^, Hypertension, Ischemic stroke, Diabetes, Liver disease, Renal failure, Malignancy, Bleeding, Gastrointestinal bleeding ^d^, Gastrointestinal bleeding ^e^, Intracranial bleeding, Urinary tract bleeding and Airway bleeding) were defined before index AF (including index admission date) were defined 2 years before index AF (including index admission date).

b: Ischemic heart disease was defined as having treatments of treadmill exercise and coronary angioplasty, or nuclear medicine image and coronary angioplasty.

c: Included hospitalized patients only.

d: Gastrointestinal bleeding was defined as having operations of panendoscopy.

e: Non-specific type of operations on gastrointestinal bleeding.

f: Previous antithrombotic treatment was defined 90 days before index AF (including index admission date).

**Fig 1:**
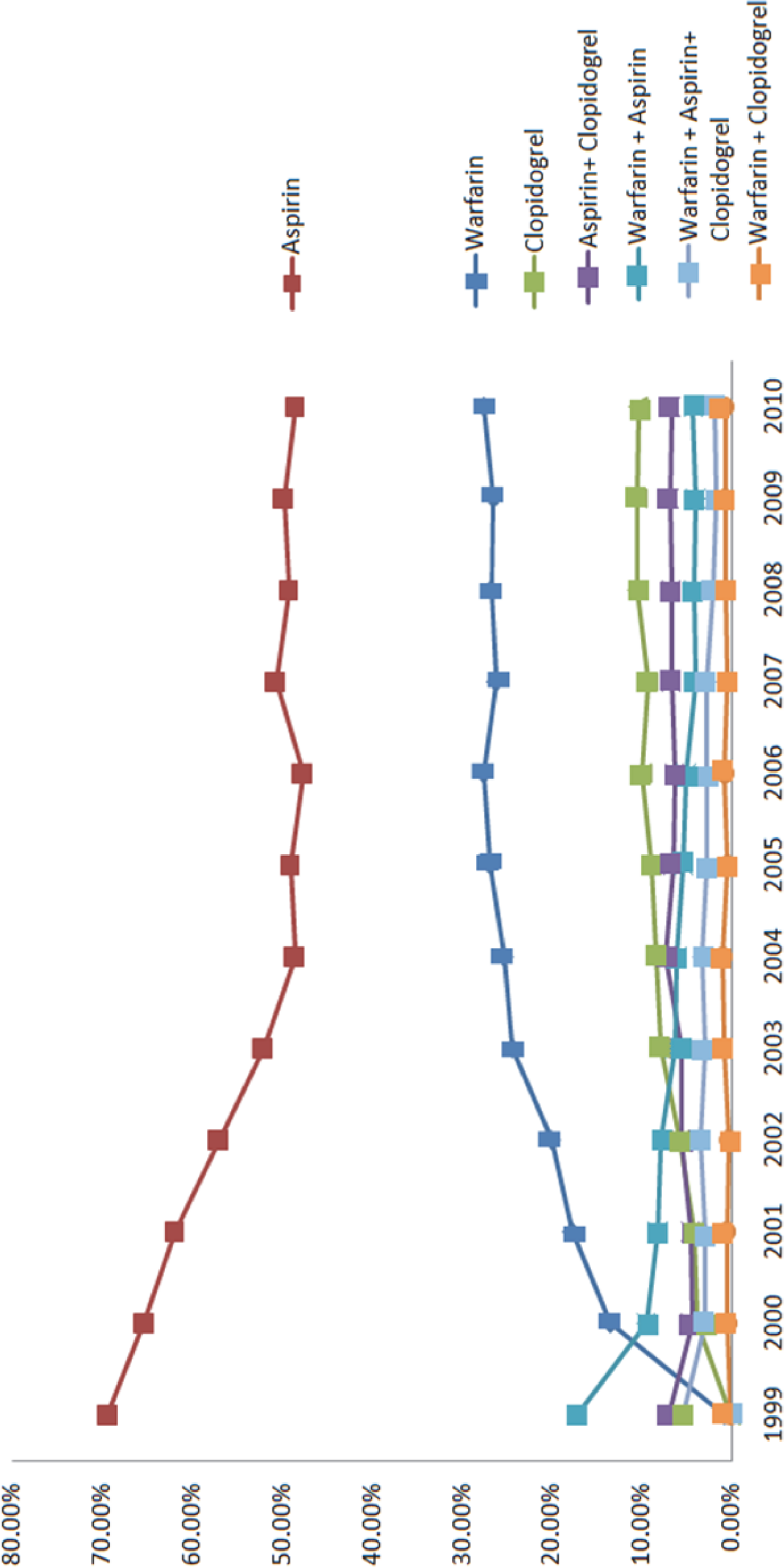
Trends of various antiplatelet and anticoagulant agent usages during the study period, 1999–2010.

Trends over time of the various antithrombotic drug used between 1999 and 2010 are shown in Fig 1. As time progressed, warfarin usage increased significantly, from 13.5% in 2000 to 27.6% in 2010. Aspirin usage decreased between 2000 (65.4%) and 2010 (48.5%). Dual and triple therapy use remained relatively stable across the time period.

**Fig 2:**
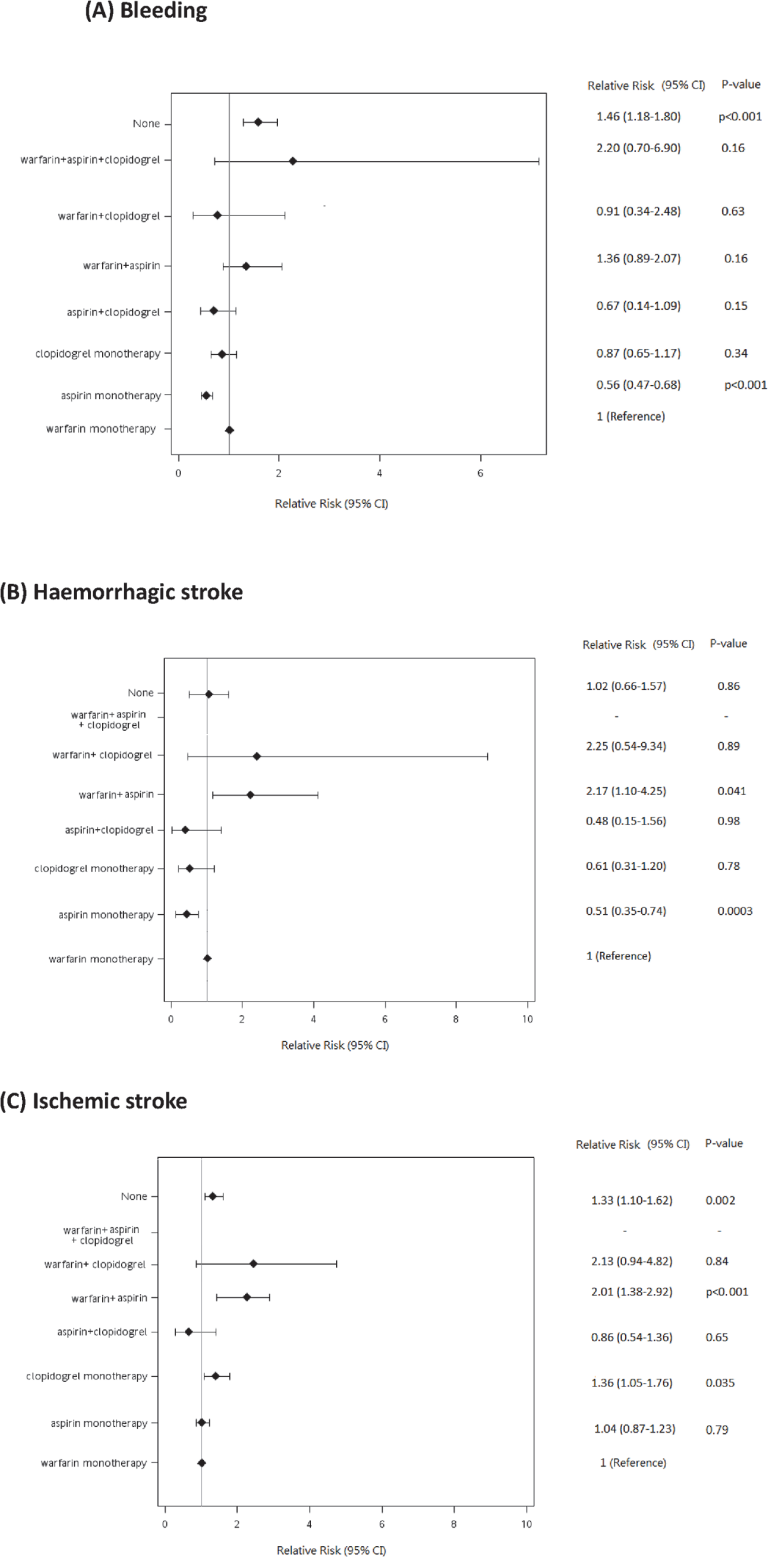
Relative risks for the risk of bleeding (A), hemorrhagic stroke (B), and ischemic stroke (C), associated with the use of warfarin, aspirin, clopidogrel, and combinations of these drugs in the study patients. CI indicates confidence interval.

During a median 3.2 (interquartile range, 1.05–6.54) years’ follow-up period, 1009 cases experienced bleeding events, whilst 224 hemorrhagic stroke and 1642 ischemic stroke events were recorded. The diagnostic image study has been performed by computed tomography (89%) and magnetic resonance image (26%). The incidence rates and associated risk estimates were listed in Table 2 and shown in Fig 2.

**Table 2:** Incidence rate and relative risks, 95% confidence intervals of events associated with anticoagulant and antiplatelet.

	Non-exposed	Antiplatelet	Warfarin and antiplatelet	Warfarin [reference]
**Bleeding**				
No. of events	261	582	33	133
Person-years of follow-up	5800	28333	745	4672
Incidence rate/1000 person-years	45.00	20.54	44.32	28.47
Relative risk (95% confidence interval), p value				
Unadjusted	1.63(1.32–2.02), 0.001	0.86(0.71–1.31),0.15	1.88(1.27–2.78), 0.043	1
Adjusted, Model 1^a^	1.45(1.17–1.80), <0.001	0.59(0.49–0.71), <0.001	1.33(0.91–1.94), 0.20	1
**Hemorrhagic stroke**				
No. of events	51	123	13	37
Person-years of follow-up	5883	29465	778	4805
Incidence rate/1000 person-years	8.67	4.17	16.71	7.70
Relative risk (95% confidence interval), p value				
Unadjusted	1.03(0.67–1.59), 0.86	0.53(0.37–0.77), <0.001	2.09(1.11–3.93), 0.031	1
Adjusted, Model 1^a^	1.02(0.66–1.57), 0.86	0.52(0.36–0.75), <0.001	2.03(1.08–3.83), 0.011	1
**Ischemic stroke**				
No. of events	370	1085	40	147
Person-years of follow-up	6239	26367	516	3942
Incidence rate/1000 person-years	59.30	41.15	77.58	37.29
Relative risk (95% confidence interval), p value				
Unadjusted	1.36(1.12–1.65), 0.003	1.08(0.91–1.27), 0.50	1.97(1.39–2.79), <0.001	1
Adjusted, Model 1^a^	1.33(1.09–1.61), 0.003	1.05(0.89–1.25), 0.50	1.90(1.34–2.70), <0.001	1

Bleeding: Model 1 was adjusted for age, sex, comorbidities (Ischemic heart, Hypertension, Ischemic stroke, Heart Failure, Diabetes, Liver disease, Renal failure, Malignancy, Bleeding, Aspirin, Warfarin and Clopidogrel) were defined before index AF (including index admission date).

Hemorrhagic stroke: Model 1 was adjusted for sex, comorbidities (Ischemic heart, Hypertension, Ischemic stroke, Liver disease, Renal failure, Bleeding, Aspirin, Warfarin and Clopidogrel) were defined before index AF (including index admission date).

Ischemic stroke: Model 1 was adjusted for sex, comorbidities (Ischemic heart, Hypertension, Ischemic stroke, Heart Failure, Diabetes, Liver disease, Renal failure, Malignancy, Aspirin, Warfarin and Clopidogrel) were defined before index AF (including index admission date).

Comorbidity was defined as presence if the disease was diagnosed two years before the index date.

### Relation to risk of bleeding and hemorrhagic stroke and therapy

Compared with those treated with warfarin (reference treatment), patients on antiplatelet drugs had a lower risk of bleeding (adjusted RR, 0.59, 95% CI, 0.49–0.71, p<0.001) and hemorrhagic stroke (RR, 0.52, 95% CI, 0.36–0.75, p<0.001) [Table 2]. Patients with combination therapy had a non-statistically significant higher risk of bleeding (RR, 1.33, 95%, 0.91–1.94, p = 0.20) and an appreciably elevated risk of hemorrhagic stroke (RR, 2.03, 95% CI, 1.08–3.83, p = 0.011). Patients taking no antithrombotic treatment had a higher risk of bleeding (RR, 1.45, 95% CI, 1.17–1.80, p<0.001), whereas no difference in incidence of hemorrhagic stroke was seen between warfarin group and no antithrombotic therapy group.

Table 3 shows results of analyses stratified by the HAS-BLED score. As expected, the bleeding risk increased with increasing HAS-BLED score. Patients on antiplatelet drugs were at a consistently lower risk for bleeding compared to the warfarin group irrespective of HAS-BLED strata (p values in each strata, 0.002 for low risk; <0.001 for intermediate risk; 0.004 for high risk). RRs of bleeding was 0.37 (95% CI, 0.19–0.69, p = 0.002) for the low risk stratum, and 0.70(95% CI, 0.55–0.89, p = 0.004) for the high risk stratum. Compared to the warfarin group, patients in the intermediate and high bleeding risk strata who were taking no antithrombotic drugs were at higher risk for bleeding (RR, 2.44, 95% CI, 1.84–3.23, p<0.001 for HAS-BLED ≥3).

**Table 3:** Incidence rate and relative risks, 95% confidence intervals of events associated with anticoagulant and antiplatelet within different risk strata.

	Non-exposed				Antiplatelet				Warfarin and antiplatelet		Warfarin [reference]	
	No. of event (incidence^*^)			RR (95%CI), P-value	No. of event (incidence^*^)			RR (95%CI), P-value	No. of event (incidence^*^)	RR (95%CI), P-value	No. of event (incidence^*^)	RR (95%CI)
**Categorization of Bleeding risk by HAS-BLED**												
Low (0–1)	38(11.9)		0.84(0.47–1.50), 0.56		21(4.8)		0.37(0.19–0.69), 0.002		3(24.7)	1.78(0.52–6.07), 0.36	17(12.5)	1.0
Intermediate (2)	78(63.2)		1.85(1.26–2.74), 0.002		144(16.9)		0.54(0.38–0.78), <0.001		6(31.6)	1.03(0.44–2.43), 0.95	39(30.5)	1.0
High (> = 3)	145(104.7)		2.44(1.84–3.23), <0.001		417(27.0)		0.70(0.55–0.89), 0.004		24(55.4)	1.43(0.91–2.27), 0.12	77(37.9)	1.0
**Categorization of risk of ischemic stroke**												
**CHADS**_**2**_ **, classical**												
Low (0)	52(18.7)	0.63(0.41–0.98), 0.034			140(21.0)	0.81(0.56–1.17), 0.25			8(69.9)	2.62(1.21–5.63), <0.001	36(25.3)	1.0
Intermediate (1–2)	124(60.4)	1.58(1.13–2.19), 0.007			428(38.3)	1.14(0.85–1.53), 0.37			11(130.2)	3.75(1.36–10.38), 0.011	51(33.5)	1.0
High (>2)	194(110.5)	1.64(1.23–2.18), <0.001			517(60.4)	0.98(0.76–1.27), 0.89			21(200.3)	2.91(1.06–7.99), 0.039	60 (66.2)	1.0
**CHADS**_**2**_ **2, revised**												
Low (0)	52(18.7)	0.63(0.41–0.98), 0.034			140(21.0)	0.81(0.56–1.17), 0.25			8(69.9)	2.62(1.21–5.63), <0.001	36(25.3)	1.0
Intermediate (1)	53 (44.9)	1.32(0.85–2.05), 0.22			221(32.7)	0.98(0.68–1.41), 0.90			6(47.5)	1.39(0.19–10.20), 0.74	33(33.9)	1.0
High (> = 2)	265(109.4)	1.66(1.29–2.12), <0.001			724(59.1)	1.07(0.86–1.34), 0.55			26(220.5)	3.94(1.82–8.52), <0.001	78(54.8)	1.0
**CHA**_**2**_ **DS**_**2**_ **-VASc**												
Low (0)	18(15.3)	0.72(0.35–1.50), 0.30			40(13.9)	0.75(0.40–1.40), 0.28			3(46.1)	2.57(0.73–9.04), 0.087	13(18.3)	1.0
Intermediate (1)	35(20.9)	0.62(0.36–1.06), 0.10			107(23.4)	0.82(0.52–1.28), 0.50			6(82.7)	2.74(1.11–6.73), 0.021	23(28.1)	1.0
High (>1)	317(78.7)	1.62(1.30–2.02), <0.001			938(51.3)	1.07(0.88–1.31), 0.63			31(84.2)	1.69(1.14–2.52), <0.001	111(47.5)	1.0

Abbreviations: CI, confidence interval; RR, relative risk.

*Incidence rate/1000 person-years

The detailed breakdown by actual therapy shown in Fig 2 shows that compared to warfarin (reference), aspirin was associated with less bleeding. However, bleeding risk was not significantly different for other categories of drug exposures.

### Relation to risk of ischemic stroke and therapy

When compared with patients on warfarin, those patients on antiplatelet drugs had a similar risk (RR 1.05, 95% CI, 0.89–1.25, p = 0.50), whilst the no antithrombotic therapy (RR 1.33, 95%CI, 1.09–1.61, p = 0.003) and combination therapy had a significantly higher risk (RR 1.90, 95% CI, 1.34–2.70, p<0.001) [Table 2].

As expected, there was increasing stroke risk with increasing risk strata of the CHADS_2_ and CHA_2_DS_2_-VASc scores [Table 3]. For the low risk strata based on the CHA_2_DS_2_-VASc score, patients taking no antithrombotic therapy or antiplatelet drugs were at similar risk for ischemic stroke, when compared to those on warfarin as the reference treatment (RR, 0.75, 95% CI, 0.40–1.40, p = 0.28 for antiplatelet drugs, RR, 0.72, 95% CI, 0.35–1.50, p = 0.30 for no therapy). For the high risk strata, patients taking no antithrombotic therapy and on combination therapy had a higher risk for ischemic stroke, compared with those on warfarin (RR, 1.62, 95% CI, 1.30–2.02, p<0.001 for no therapy; RR, 1.69, 95% CI, 1.14–2.52, p<0.001 for combination therapy); however, patients taking antiplatelet drugs had a similar risk for ischemic stroke (RR, 1.07, 95% CI, 0.88–1.31, p = 0.63). A similar pattern was evident when analyzed by the CHADS_2_ score (classical or revised).

The detailed breakdown by actual therapy shown in Fig 2 shows that compared to warfarin (reference treatment), the use of aspirin and aspirin+clopidogrel had similar risk of ischemic stroke, whilst stroke risk was increased with warfarin+aspirin, with a trend for warfarin+clopidogrel.

## Discussion

In this study of a large national representative cohort of AF patients from Taiwan, we show for the first time that antiplatelet therapy had no significant difference in ischemic stroke risk to warfarin, whilst combination therapy was associated with higher risks. For hemorrhagic stroke and bleeding, aspirin had a lower risk compared to warfarin, whilst combination therapy conferred a higher risk. These data are consistent with the poor efficacy and safety of warfarin in Asian patients, and offers important opportunities for improved stroke prevention with novel OACs.

A metaanalysis of clinical trial data has shown that adjusted dose anticoagulant therapy compared to control reduces stroke in AF by 64% and all cause mortality by 26%, and that warfarin was more protective than aspirin[21]. However, uncertain factors, such as genetics, dietary and drug factors make the adjustment of warfarin dosage unpredictable[17]. Our study clearly demonstrates antiplatelet usage had a lower risk for bleeding and hemorrhagic stroke, compared with warfarin, consistent with older trial data in Western population[21].

Interestingly we found that patients without any antithrombotic therapy in the high risk strata had a higher risk for bleeding, as well as ischemic stroke, compared to warfarin use. This is inconsistent with large observational data in USA and Swedish cohorts where compared with no warfarin use, patients treated with warfarin had a lower risk for ischemic stroke and hemorrhage[22–24]. Residual confounding may explain this, representing associated comorbidities, or risk factors for bleeding leading to non-prescription of OAC.

A population-based elderly cohort based on 125195 elderly patients (> = 66 years old) with AF in Ontario showed the warfarin therapy was associated an overall rate of 3.8 per 100 person-year for bleeding, and the rates of bleeding increased from 1.8 in the low stratum of CHADS_2_ to 6.7 per 100 person-year in the CHADS_2_ scores of 4 or greater[25]. Our results showed similar bleeding event rates by HAS-BLED scores, which has recently been shown to be a better predictor for serious bleeding compared to the CHADS_2_ and CHA_2_DS_2_-VASc scores[26,27].

Importantly, racial difference for drug susceptibility may be evident, especially for warfarin. Indeed, non-white patients had a higher risk for warfarin-related hemorrhagic stroke risk, and Asian population in particular have a higher risk for warfarin-related intracranial hemorrhage (relative risk,4.06, 95% CI, 2.47–6.65)[28]. Also, Asian patients with AF may do badly on warfarin compared to non-Asian patients, with higher rates of stroke, hemorrhagic stroke, major bleeding and intracranial bleeding[29]. Our data are also consistent with a small study by Guo et al[19], which showed antiplatelet drugs and warfarin had a similar risk of stroke/thromboembolism.

Nonetheless, efficacy and safety whilst on warfarin is highly dependent upon the quality of anticoagulation control, as reflected by average time in therapeutic range (TTR)[15,16,30]. For warfarin, this is established as an INR of 2.0–3.0, and an average TTR of >70% is recommended in guidelines[31]. Unfortunately, our dataset does not have detailed TTR data, but we know from other published studies that the average TTR for warfarin is low, for example, being only 44% in the RE-LY trial[32]. Also, there is the perception that an INR 1.6–2.6 is best for older patients, and this may contribute to the stroke rates seen on warfarin, which appears no different to those on aspirin[33]. Herbal medicines are commonly used in our population which may also influence TTR status[34]. Nonetheless, our study shows a progressive increase in use of warfarin among the Taiwanese patients over time, although usage rates are still low when compared with Danish cohort (36.6%)[23].

The strengths of our study included a large population-based follow-up study and the integrated details of prescription records such as the drug used, dosages, days of supply dispensed from database. The health insurance program in Taiwan covers more than 99% of the adult population, and the cohort was a representative sample of population of Taiwan. Several studies based on the cohort showed that the score systems of CHADS_2_ and CHA_2_DS_2_-VASc were applicable in Taiwan[8,35], and the results were compatible with a community based cohort [5,36].

### Limitations

First, the history of AF prior to 1996 was unknown, but patients with any record of AF before 1999 were excluded to reduce the possibility of including prevalent cases. Second, the validity of diagnosis of bleeding and stroke events may influence on our results, even though the accuracy of recording stroke diagnoses and prescriptions in NHIRD was high. Third, we lacked data for lifestyle information such as weight, drinking, or smoking status in this cohort. Finally, no data of adherence to the drug usage was obtained, nor TTR data, even we used time dependent covariate model to handle the exposure status. Poor adherence with various drug treatments may confound the estimation of bleeding and stroke risk.

In conclusion, in this study of a large national representative cohort from Taiwan, we show for the first time that antiplatelet therapy had no significant difference in ischemic stroke risk to warfarin, whilst for bleeding, aspirin had a lower risk compared to warfarin. This may reflect poor anticoagulation control, highlighting opportunities for improved stroke prevention with alternative strategies, such as the novel OACs.

## Supporting Information

S1 Table ICD-9-CM codes for comorbidities. (DOCX)

S2 Table CHADS2, CHA2DS2-VASc and HAS-BLED score definition. (DOCX)

S3 Table Number and crude Incidence rate of bleeding by the HAS-BLED score as well as ischemic stroke by the CHADS2 and CHA2DS2-VASc score among patients in the prescription groups. (DOCX)

## References

[1] Friberg L, Rosenqvist M, Lip GY. Evaluation of risk stratification schemes for ischaemic stroke and bleeding in 182 678 patients with atrial fibrillation: the Swedish Atrial Fibrillation cohort study. Eur Heart J. 2012; 33: 1500–1510. 10.1093/eurheartj/ehr488 22246443

[2] Lee CH, Liu PY, Tsai LM, Tsai WC, Ho MT, Chen JH, et al Characteristics of hospitalized patients with atrial fibrillation in Taiwan: a nationwide observation. Am J Med. 2007;120: 819 e811-817. 17765053 10.1016/j.amjmed.2006.10.014

[3] Potpara TS, Lip GYH. Ischemic Stroke and Atrial Fibrillation—A DeadlySerious Combination. Cerebrovascular Diseases. 2011; 32: 461–462. 10.1159/000332030 22005454

[4] Chien KL, Su TC, Hsu HC, Chang WT, Chen PC, Sung FC, et al Constructing the prediction model for the risk of stroke in a Chinese population: report from a cohort study in Taiwan. Stroke. 2010; 41: 1858–1864. 10.1161/STROKEAHA.110.586222 20671251

[5] Chien KL, Su TC, Hsu HC, Chang WT, Chen PC, Chen MF, et al Atrial fibrillation prevalence, incidence and risk of stroke and all-cause death among Chinese. Int J Cardiol. 2010; 139: 173–180. 10.1016/j.ijcard.2008.10.045 19046608

[6] Gage BF, Waterman AD, Shannon W, Boechler M, Rich MW, Radford MJ. Validation of clinical classification schemes for predicting stroke: results from the National Registry of Atrial Fibrillation. JAMA. 2001; 285: 2864–2870. 11401607 10.1001/jama.285.22.2864

[7] Lip GY, Nieuwlaat R, Pisters R, Lane DA, Crijns HJ. Refining clinical risk stratification for predicting stroke and thromboembolism in atrial fibrillation using a novel risk factor-based approach: the euro heart survey on atrial fibrillation. Chest. 2010; 137: 263–272. 10.1378/chest.09-1584 19762550

[8] Lin LY, Lee CH, Yu CC, Tsai CT, Lai LP, Hwang JJ, et al Risk factors and incidence of ischemic stroke in Taiwanese with nonvalvular atrial fibrillation—a nation wide database analysis. Atherosclerosis. 2011; 217: 292–295. 10.1016/j.atherosclerosis.2011.03.033 21513938

[9] Friberg L, Benson L, Rosenqvist M, Lip GY. Assessment of female sex as a risk factor in atrial fibrillation in Sweden: nationwide retrospective cohort study. BMJ. 2012; 344: e3522 10.1136/bmj.e3522 22653980 PMC3365143

[10] Olesen JB, Torp-Pedersen C, Hansen ML, Lip GY. The value of the CHA2DS2-VASc score for refining stroke risk stratification in patients with atrial fibrillation with a CHADS2 score 0–1: a nationwide cohort study. Thromb Haemost 2012; 107: 1172–1179. 10.1160/TH12-03-0175 22473219

[11] Hart RG, Benavente O, McBride R, Pearce LA. Antithrombotic therapy to prevent stroke in patients with atrial fibrillation: a meta-analysis. Ann Intern Med. 1999; 131: 492–501. 10507957 10.7326/0003-4819-131-7-199910050-00003

[12] Lip GY, Andreotti F, Fauchier L, Huber K, Hylek E, Knight E. Executive Summary of a Position Document from the European Heart Rhythm Association [EHRA], endorsed by the European Society of Cardiology [ESC] Working Group on Thrombosis. Thromb Haemost. 2011; 106: 997–1011. 10.1160/TH11-10-0690 22048796

[13] Hansen ML, Sørensen R, Clausen MT, Fog-Petersen ML, Raunsø J, Gadsbøll N, et al Risk of bleeding with single, dual, or triple therapy with warfarin, aspirin, and clopidogrel in patients with atrial fibrillation. Arch Intern Med. 2010; 170: 1433–1441. 10.1001/archinternmed.2010.271 20837828

[14] Röther J, Crijns H. Prevention of Stroke in Patients with Atrial Fibrillation: The Role of New Antiarrhythmic and Antithrombotic Drugs. Cerebrovascular Diseases. 2010; 30: 314–322. 10.1159/000319608 20664267

[15] Gallagher AM, Setakis E, Plumb JM, Clemens A, van Staa TP. Risks of stroke and mortality associated with suboptimal anticoagulation in atrial fibrillation patients. Thromb Haemost. 2011; 106: 968–977. 10.1160/TH11-05-0353 21901239

[16] Wan Y, Heneghan C, Perera R, Roberts N, Hollowell J, Glasziou P, et al Anticoagulation control and prediction of adverse events in patients with atrial fibrillation: a systematic review. Circ Cardiovasc Qual Outcomes 2008; 1: 84–91. 10.1161/CIRCOUTCOMES.108.796185 20031794

[17] De Caterina R, Husted S, Wallentin L, Andreotti F, Arnesen H, Bachmann F, et al New oral anticoagulants in atrial fibrillation and acute coronary syndromes: ESC Working Group on Thrombosis-Task Force on Anticoagulants in Heart Disease position paper. J Am Coll Cardiol. 2012; 59: 1413–1425. 10.1016/j.jacc.2012.02.008 22497820

[18] Wallentin L, Yusuf S, Ezekowitz MD, Alings M, Flather M, Franzosi MG, et al Efficacy and safety of dabigatran compared with warfarin at different levels of international normalised ratio control for stroke prevention in atrial fibrillation: an analysis of the RE-LY trial. Lancet. 2010; 376: 975–983. 10.1016/S0140-6736(10)61194-4 20801496

[19] Guo Y, Pisters R, Apostolakis S, Blann AD, Wang H, Zhao X, et al Stroke risk and suboptimal thromboprophylaxis in Chinese patients with atrial fibrillation: Would the novel oral anticoagulants have an impact? Int J Cardiol. 2013; 168: 515–522. 10.1016/j.ijcard.2012.09.187 23103146

[20] Tsai WC, Chen CY, Kuo HF, Wu MT, Tang WH, Chu CS, et al Areca nut chewing and risk of atrial fibrillation in Taiwanese men: a nationwide ecological study. Int J Med Sci. 2013; 10: 804–811. 10.7150/ijms.5998 23794944 PMC3689880

[21] Hart RG, Pearce LA, Aguilar MI. Meta-analysis: antithrombotic therapy to prevent stroke in patients who have nonvalvular atrial fibrillation. Ann Intern Med. 2007; 146: 857–867. 17577005 10.7326/0003-4819-146-12-200706190-00007

[22] Friberg L, Rosenqvist M, Lip GY. Net clinical benefit of warfarin in patients with atrial fibrillation: a report from the Swedish atrial fibrillation cohort study. Circulation. 2012; 125: 2298–2307. 10.1161/CIRCULATIONAHA.111.055079 22514252

[23] Olesen JB, Lip GY, Lindhardsen J, Lane DA, Ahlehoff O, Hansen ML, et al Risks of thromboembolism and bleeding with thromboprophylaxis in patients with atrial fibrillation: A net clinical benefit analysis using a 'real world' nationwide cohort study. Thromb Haemost. 2011; 106: 739–749. 10.1160/TH11-05-0364 21789337

[24] Singer DE, Chang Y, Fang MC, Borowsky LH, Pomernacki NK, Udaltsova N, et al The net clinical benefit of warfarin anticoagulation in atrial fibrillation. Ann Intern Med. 2009; 151: 297–305. 19721017 10.7326/0003-4819-151-5-200909010-00003 PMC2777526

[25] Gomes T, Mamdani MM, Holbrook AM, Paterson JM, Hellings C, Juurlink DN. Rates of hemorrhage during warfarin therapy for atrial fibrillation. CMAJ. 2013; 185: E121–127. 10.1503/cmaj.121218 23184840 PMC3563912

[26] Roldán V, Marín F, Manzano-Fernández S, Gallego P, Vílchez JA, Valdés M. The HAS-BLED score has better prediction accuracy for major bleeding than the CHADS or CHADS-VASc scores In anticoagulated patients with atrial fibrillation. J Am Coll Cardiol. 2013; 62:2199–204. 10.1016/j.jacc.2013.08.1623 24055744

[27] Apostolakis S, Lane DA, Buller H, Lip GY. Comparison of the CHADS2, CHA2DS2-VASc and HAS-BLED scores for the prediction of clinically relevant bleeding in anticoagulated patients with atrial fibrillation: The AMADEUS trial. Thromb Haemost. 2013; 110: 1074–1075. 10.1160/TH13-07-0552 24048467

[28] Shen AY, Yao JF, Brar SS, Jorgensen MB, Chen W. Racial/ethnic differences in the risk of intracranial hemorrhage among patients with atrial fibrillation. J Am Coll Cardiol. 2007; 50: 309–315. 17659197 10.1016/j.jacc.2007.01.098

[29] Hori M, Connolly SJ, Zhu J, Liu LS, Lau CP, Pais P, et al Dabigatran versus warfarin: effects on ischemic and hemorrhagic strokes and bleeding in Asians and non-Asians with atrial fibrillation. Stroke. 2013; 44: 1891–1896. 10.1161/STROKEAHA.113.000990 23743976

[30] Morgan CL, McEwan P, Tukiendorf A, Robinson PA, Clemens A, Plumb JM. Warfarin treatment in patients with atrial fibrillation: observing outcomes associated with varying levels of INR control. Thromb Res. 2009; 124: 37–41. 10.1016/j.thromres.2008.09.016 19062079

[31] Camm AJ, Lip GY, De Caterina R, Savelieva I, Atar D, Hohnloser SH. 2012 focused update of the ESC Guidelines for the management of atrial fibrillation: an update of the 2010 ESC Guidelines for the management of atrial fibrillation. Developed with the special contribution of the European Heart Rhythm Association. Eur Heart J. 2012; 33: 2719–2747. 10.1093/eurheartj/ehs253 22922413

[32] Wallentin L, Becker RC, James SK, Harrington RA. The PLATO trial reveals further opportunities to improve outcomes in patients with acute coronary syndrome. Editorial on Serebruany. "Viewpoint: Paradoxical excess mortality in the PLATO trial should be independently verified" (Thromb Haemost 2011; 105.5). Thromb Haemost. 2011; 105: 760–762. 10.1160/TH11-03-0162 21394383

[33] Ogawa S, Aonuma K, Tse H-F, Huang D, Huang J-L, Kalman J, et al The APHRS's 2013 statement on antithrombotic therapy of patients with nonvalvular atrial fibrillation. Journal of Arrhythmia. 2013; 29: 190–200.

[34] Wong RS, Cheng G, Chan TY. Use of herbal medicines by patients receiving warfarin. Drug Saf. 2003; 26: 585–588. 12825970 10.2165/00002018-200326080-00004

[35] Chao TF, Liu CJ, Chen SJ, Wang KL, Lin YJ, Chang SL, et al Atrial Fibrillation and the Risk of Ischemic Stroke. Stroke. 2012; 43: 2551–2555. 22871677 10.1161/STROKEAHA.112.667865

[36] Lip GY, Rasmussen LH, Skjoth F, Overvad K, Larsen TB. Stroke and mortality in patients with incident heart failure: the Diet, Cancer and Health (DCH) cohort study. BMJ Open. 2012; 2. 10.1136/bmjopen-2012-000975 PMC4400696 22773537

