# Supplementary material for: Risk of Bleeding and Stroke with Oral Anticoagulation and Antiplatelet Therapy in Patients with Atrial Fibrillation in Taiwan: A Nationwide Cohort Study
Source: PLoS One. 2015 Apr 29;10(4):e0125257. doi: 10.1371/journal.pone.0125257 (PMC4414564; doi:10.1371/journal.pone.0125257)
Supplement: S1 Table — (DOCX) [file pone.0125257.s001.docx]

**S1 Table. ICD-9-CM codes for comorbidities**

| CODE | CATEGORY | NAME |
| --- | --- | --- |
| 4273 | Patient inclusion | Atrial Fibrillation |
| 393-398 | Patient exclusion | Rheumatic Heart Disease |
| 410 | comorbidity | Acute myocardial infarction |
| 410-414 | comorbidity | Ischemic heart disease |
| 18015B | comorbidity | Treadmill exercise test |
| 18022B | comorbidity | Coronary angioplasty |
| 26024A, 26024B, 26025A, 26025B,  26069B | comorbidity | Nuclear medicine image |
| 428 | comorbidity | Heart failure |
| 7463, 74722, 4241, 7465, 4240, 4241 | comorbidity | Valvular heart disease |
| 680168A  680168B | comorbidity | mitral valve plasty |
| 401-405 | comorbidity | Hypertension |
| 433-438 | comorbidity | Ischemic stroke |
| 250 | comorbidity | Diabetes |
| 570-573 | comorbidity | Liver disease |
| 584-585 | comorbidity | Renal failure |
| 140-208 | comorbidity | Malignancy |
| 578 | comorbidity | Gastrointestinal bleeding |
| 28016C | comorbidity | Panendoscopy |
| 430-432 | comorbidity | Intracranial bleeding |
| 5997 | comorbidity | Urinary tract bleeding: hematuria |
| 7863 | comorbidity | Airway bleeding: hemoptysis  Bleeding |
