# Supplementary material for: Risk of Bleeding and Stroke with Oral Anticoagulation and Antiplatelet Therapy in Patients with Atrial Fibrillation in Taiwan: A Nationwide Cohort Study
Source: PLoS One. 2015 Apr 29;10(4):e0125257. doi: 10.1371/journal.pone.0125257 (PMC4414564; doi:10.1371/journal.pone.0125257)
Supplement: S2 Table — (DOCX) [file pone.0125257.s002.docx]

**S2 Table. CHADS2, CHA2DS2-VASc and HAS-BLED score definition**

| Name | ICD 9 code | Score |
| --- | --- | --- |
| CHA2DS2-VASc |  | **0-9** |
| Congestive heart failure/LV dysfunction | 1. (hospitalization) | 1 |
| Hypertension | 401-405 | 1 |
| Age, year |  |  |
| >=75 |  | 2 |
| 65-74 |  | 1 |
| Diabetes mellitus | 250 | 1 |
| Stroke/TIA/thromboembolism | 433-438 | 2 |
| Vascular disease | Prior myocardial infarction (410.0-410.9;411.0;412;429.79), or Peripheral artery disease (440.20-420.24;420.29;440.30-440.32; 440.4;443.81;443.89;443.9; 440.20-440.24;440.29;440.3;440.4), or Aortic plaque (440.0) | 1 |
| Sex category | Female=1; male=0 | 1 |
| CHADS2 |  | **0-6** |
| Congestive heart failure | 1. (hospitalization) | 1 |
| Hypertension | 401-405 | 1 |
| Age>=75 yr |  | 1 |
| Diabetes mellitus | 250 | 1 |
| Stroke/TIA/thromboembolism | 433-438 | 2 |
| HAS-BLED |  | **0-7** |
| Hypertension | 401-405 | 1 |
| Abnormal renal and liver function (one point each) | 582,583,585,586,570-573,790.4 | 1. or 2 |
| Stroke | 433-438 | 1 |
| Bleeding | 578,430,431,432,5997,7863 | 1 |
| Labile INRs | Not available |  |
| Elderly (age >65 yr) |  | 1 |
| Drug | Antiplatelet agents and non-steroidal anti-inflammatory drug (information on alcohol is not available) | 1 |
