# Supplementary material for: Risk of Bleeding and Stroke with Oral Anticoagulation and Antiplatelet Therapy in Patients with Atrial Fibrillation in Taiwan: A Nationwide Cohort Study
Source: PLoS One. 2015 Apr 29;10(4):e0125257. doi: 10.1371/journal.pone.0125257 (PMC4414564; doi:10.1371/journal.pone.0125257)
Supplement: S3 Table — (DOCX) [file pone.0125257.s003.docx]

**S3 Table. Number and crude Incidence rate of bleeding by the HAS-BLED score as well as ischemic stroke by the CHADS2 and CHA2DS2-VASc score among patients in the prescription groups**

| **Risk Score** | **All** | |  | | **Warfarin-based** |  | | **Aspirin or clopidogrel** | | |  | | **Non-exposed** |  |
| --- | --- | --- | --- | --- | --- | --- | --- | --- | --- | --- | --- | --- | --- | --- |
|  | **n (incidence rate)** | | **Relative Risk (95% CI)** | | **n (incidence rate)** | **Relative Risk (95% CI)** | | **n (incidence rate)** | | | **Relative Risk (95% CI)** | | **n (incidence rate)** | **Relative Risk (95% CI)** |
| **Categorization of Bleeding risk** |  |  | |  | |  | |  | |  | |  | |  |
| **HAS-BLED** |  |  | |  | |  | |  | |  | |  | |  |
| 0 | 7(0.37) | 1.0 | | 2(0.71) | | 1.0 | | 0(0) | | - | | 5(0.31) | | 1.0 |
| 1 | 72(1.02) | 2.69(1.2-5.85) | | 18(1.50) | | 2.11(0.49-9.09) | | 21(0.49) | | 1.0 | | 33(2.11) | | 5.84(2.27-15.00) |
| 2 | 267(2.38) | 6.21(2.93-13.15) | | 45(3.07) | | 4.38(1.06-18.05) | | 144(1.69) | | 3.47(2.20-5.49) | | 78(6.32) | | 14.86(6.00-36.81) |
| 3 | 332(2.66) | 6.82(3.23-14.42) | | 63(3.96) | | 5.67(1.39-23.19) | | 213(2.12) | | 4.34(2.77-6.79) | | 56(6.69) | | 12.93(5.14-32.53) |
| 4 | 236(4.23) | 10.72(5.05-22.75) | | 27(3.38) | | 4.85(1.15-20.41) | | 146(3.38) | | 6.89(4.36-10.90) | | 63(13.96) | | 27.65(11.06-69.11) |
| 5 | 78(7.05) | 17.23(7.94-37.36) | | 10(17.94) | | 26.45(5.77-121.25) | | 48(4.94) | | 10.03(6.00-16.77) | | 20(25.53) | | 39.67(14.77-106.56) |
| 6 | 16(10.96) | 25.72(10.57-62.57) | | 1(5.25) | | 7.84(0.71-87.13) | | 10(9.18) | | 18.28(8.60-38.85) | | 5(27.63) | | 42.61(12.25-148.20) |
| 7 | 1(20.45) | 42.49(5.24-344.33) | | 0(0) | | - | | 0(0) | | - | | 1(98.18) | | 103.24(11.99-889.07) |
| **Categorization of risk of ischemic stroke** |  |  | | |  |  | |  | | |  | |  |  |
| **CHADS_2_** |  |  | | |  |  | |  | | |  | |  |  |
| 0 | 236 (2.15) | 1.0 | | | 44 (2.86) | 1.0 | | 140 (2.10) | | | 1.0 | | 52 (1.87) | 1.0 |
| 1 | 308 (3.49) | 1.63(1.37-1.93) | | | 34 (3.39) | 1.16(0.74-1.83) | | 221 (3.27) | | | 1.55(1.25-1.91) | | 53 (4.96) | 2.54(1.69-3.80) |
| 2 | 298 (5.00) | 2.21(1.86-2.63) | | | 21 (3.83) | 1.25(0.74-2.11) | | 206 (4.66) | | | 2.15(1.72-2.66) | | 71 (7.20) | 3.30(2.25-4.81) |
| 3 | 366 (6.12) | 2.65(2.24-3.13) | | | 35 (6.17) | 2.03(1.29-3.19) | | 248 (5.40) | | | 2.46(2.00-3.03) | | 83(10.16) | 4.14(2.88-6.07) |
| 4 | 195 (7.78) | 3.31(2.73-4.01) | | | 30 (4.63) | 1.61(0.87-3.01) | | 116 (6.51) | | | 3.09(2.44-3.92) | | 49 (15.46) | 5.92(3.88-8.93) |
| 5 | 163(11.12) | 4.54(3.71-5.57) | | | 13 (8.52) | 2.64(1.41-4.95) | | 105 (9.14) | | | 3.99(3.09-5.15) | | 45 (25.01) | 9.20(6.01-14.05) |
| 6 | 76 (12.39) | 4.82(3.70-6.28) | | | 10 (22.66) | 6.33(3.12-12.84) | | 49 (10.52) | | | 4.51(3.25-6.25) | | 17 (10.64) | 5.57(3.08-10.05) |
| **CHA_2_DS_2_-VASc** |  | |  | |  |  |  | |  | | |  | |  |
| 0 | 74(1.53) | | 1.0 | | 16(2.06) | 1.0 | 40(1.39) | | 1.0 | | | 18(1.53) | | 1.0 |
| 1 | 171(2.39) | | 1.54(1.17-2.03) | | 29(3.26) | 1.56(0.85-2.87) | 107(2.34) | | 1.67(1.16-2.40) | | | 35(2.09) | | 1.29(0.70-2.37) |
| 2 | 312(4.05) | | 2.58(2.00-3.33) | | 32(3.42) | 1.60(0.88-2.91) | 213(3.79) | | 2.68(1.91-3.75) | | | 67(5.80) | | 3.39(1.95-5.88) |
| 3 | 354(4.87) | | 3.04(2.36-3.92) | | 39(5.31) | 2.46(1.37-4.41) | 236(4.31) | | 3.00(2.14-4.20) | | | 79(7.43) | | 4.14(2.40-7.12) |
| 4 | 313(6.39) | | 3.84(2.97-4.96) | | 29(5.17) | 2.34(1.27-4.33) | 215(5.17) | | 3.93(2.81-5.52) | | | 69(11.31) | | 5.49(3.16-9.56) |
| 5 | 206(7.62) | | 4.56(3.49-5.98) | | 26(8.19) | 3.68(1.97-6.88) | 134(6.46) | | 4.39(3.08-6.25) | | | 46(14.78) | | 6.91(3.87-12.34) |
| 6 | 149(11.64) | | 6.63(4.99-8.80) | | 11(9.02) | 3.84(1.77-8.31) | 96(9.68) | | 6.37(4.40-9.22) | | | 42(25.27) | | 10.91(6.06-19.64) |
| 7 | 49(12.05) | | 6.56(4.54-9.49) | | 5(16.62) | 6.45(2.33-17.81) | 33(10.52) | | 6.86(4.32-10.90) | | | 11(17.47) | | 6.98(3.07-15.87) |
| 8 | 13(14.75) | | 7.58(4.19-13.71) | | 0(0) | - | 10(14.38) | | 8.74(4.36-17.51) | | | 3(24.45) | | 9.97(2.89-34.39) |
| 9 | 1(27.97) | | 12.53(1.74-90.19) | | 0(0) | - | 1(147.28) | | 58.98(8.07-431.110) | | | 0(0) | | - |

Warfarin-based: warfarin monotherapy; warfarin+aspirin; warfarin+clopidogrel; warfarin+aspirin +clopidogrel.

Aspirin or clopidogrel: aspirin monotherapy; clopidogrel monotherapy; aspirin+clopidogrel.
